# Preparation of high-quality YAG:Ce^3+^ ceramic phosphor by high-frequency induction heated press sintering methods

**DOI:** 10.1038/s41598-022-23094-z

**Published:** 2022-11-28

**Authors:** Seok Bin Kwon, Jung Hyeon Yoo, Seung Hee Choi, MinYoung Na, Bo Young Kim, Seon Young Lee, Ho-Jung Jeong, Wan Ho Kim, Seoung Hyok Park, Ho Shin Yoon, Bong Kyun Kang, Young Hyun Song, Dae Ho Yoon

**Affiliations:** 1 School of Advanced Materials Science and Engineering, SungKyunKwan University, Suwon, 16419 Republic of Korea; 2 Lighting Materials and Components Research Center, Korea Photonics Technology Institute, Gwangju, 61007 Republic of Korea; 3 Force4, Gwangju, 61009 Republic of Korea; 4 Department of Electronic Materials, Devices, and Equipment Engineering, Soonchunhyang University, 22, Soonchunhyang-ro, Asan City, Chungnam 31538 Republic of Korea; 5 Department of Display Materials Engineering, Soonchunhyang University, 22, Soonchunhyang-ro, Asan City, Chungnam 31538 Republic of Korea

**Keywords:** Materials for optics, Engineering

## Abstract

This study investigates the characteristics of a ceramic phosphor (CP) for the converter of a high-power laser diode-based automobile headlamp synthesized by high-frequency induction heated press (HFP) sintering. The CP prepared by an HFP method exhibits remarkable optical properties that are comparable to spark plasma sintering. The effects of post-treatment process for controlling residual pores, as well as sintering temperature, sintering pressure and heating rate for optimization of the HFP sintering method, were studied. The HFP sintering process can be widely used in ceramics and lighting fields because it is designed relatively low cost compared to other techniques and exhibits excellent productivity.

## Introduction

The headlamp, one of the parts of a car, plays an important role in determining the role of lighting and the impression of a vehicle. A headlamp supports safe driving by facilitating the sight of the driver, and making a presence for pedestrians^1^. Due to the important role of the headlamp, it has been actively researched for decades, and currently the research trends are changing in terms of lighting and design^2–6^. First, the lighting function is researched to irradiate further distances to facilitate longer sight to the driver. Second, the high-power light source is being miniaturized to differentiate the design aspect from the others. And it is also expanding its applicability, such as intelligent headlamps. Nowadays, vehicle lighting industries have chosen the white light emitting diode (wLED) type that generates white light by converting blue light from GaN to yellow using phosphor, because of the advantages of enhancing the lifetime of the light source, its eco-friendly nature, and the reduction of energy consumption it offers^7,8^. This type of LED offers advantages over the multi-chip type that generates white light by combining multiple chips that emit different wavelengths for process simplifying, improving the luminous efficacy and facile control of the optoelectrical characteristics. However, despite those advantages, the LED has an issue of luminous ‘efficiency droop’, due to light saturation when the applied current is increased^9,10^. Efficiency droop refers to a phenomenon in which the output efficiency decreases when the input power to the LED device rises above the critical point.

One of the Cerium-doped garnet phosphors, YAG:Ce^3+^ is well known as a high-stability phosphor material and has excellent chemical stability, high quantum yield, moisture resistance, and thermal expansion properties. A composite of silicone resin and phosphor is mostly used as a light conversion material for wLED^11,12^. However, in this case, the resin material is easily damaged or burned by the heat generated from the chip, resulting in performance degradation^13,14^. To handle these issues, the remote-type chip design that makes a space between the light source and the color conversion layer has been suggested by fabricating the color conversion layer as a film or plate by mixing phosphors with silicone resin or glass materials^15–17^. While these are technical improvements, the performance decrement issues are still open to debate, because the currently required generating power standard for next-generation headlamp lighting is being increased. As an alternative to the common LED, the laser diode (LD) that has a fast-switching rate, narrow emission spectrum, and high luminous efficacy without performance droop is attracted attention as a next-generation light source^14,18^. However, because the LD is driven by strong power, the silicone and glass composite phosphors that were fabricated by the traditional method are not suitable to apply in the LD, due to the silicone being burned, and the glass composite broken^19^.

Disk-type phosphor converters, such as CP and single-crystal phosphors, which are currently being actively studied, are newly proposed methods for application to high-power LDs^14,18–22^. An important part in the manufacture of polycrystalline CP is the sintering process, and the various methods, such as vacuum sintering (VS), gas pressure sintering (GPS), and SPS, greatly affect both the quality of the material, and its commercialization. The VS and GPS sintering methods, which to date have been widely used in the manufacture of ceramics, have the advantage of being able to control a very high temperature, so that a densified sintered body can be manufactured^23–25^. However, VS and GPS sintering furnaces, which have restrictions on the rate of temperature increase, are inevitably disadvantageous in terms of their low productivity. On the other hand, the SPS sintering furnace, which is heated by passing a electric current (AC, pulse AC or DC), is equipment that can obtain a high-density sintered compound that is capable of a fast temperature increase rate^26,27^. Although SPS equipment has excellent productivity, it has the disadvantage of being made of expensive components. Therefore, it is necessary to study the potential of various other sintering methods in the field of CPs.

In this study, we manufactured high-quality CPs using high-frequency induction heated press (HFP) sintering equipment with excellent productivity. The HFP sintering is a method of manufacturing a densified sintered body by induction heating using a high-frequency principle while applying pressure to the object during the process. Compared to other heating methods, such as heat reflection and heat conduction, the HFP sintering method, which directly heats an object, offers very good thermal efficiency and a faster temperature increase rate, enabling uniform sintered body synthesis in a short time. To examine the superiority of the CP manufactured by the HFP sintering method, we prepared each CP using the SPS, GPS, and HFP methods, and compared their properties.

## Materials and method

Spherical-shaped nano-sized YAG:Ce^3+^ powders were prepared by the same method reported previously by Song et al^21^. Y(NO_3_)_3_∙6H_2_O, Ce(NO_3_)_3_∙6H_2_O, Al(NO_3_)_3_∙9H_2_O, and (NH_4_)Al(SO_4_)_2_ were dissolved in distilled water at molar concentrations of 2.95, 0.05, 2.5, and 2.5, respectively. Urea was added and reacted at 90 °C to prepare a precipitate. The obtained precipitate was calcined at 1200 °C to prepare a spherical phosphor.

### HFP-CP

**Figure 1:**
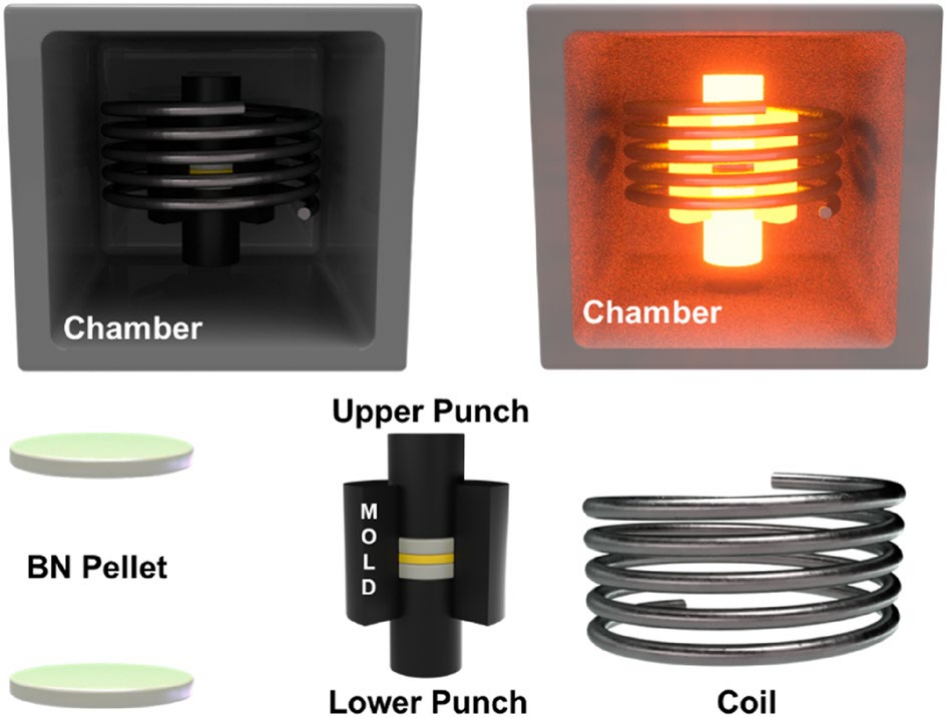
Schematic of HPF equipment configuration.

YAG:Ce^3+^ powders were put on boron nitride pellet over the carbon punch, and another BN pellet was put above them. Covered by the upper punch, samples were sintered at 1650, 1750, and 1850 °C for 10 min under 50 MPa. In the case of the sample sintered at 1750 °C, which had the best performance, the temperature increase rate was divided into 50, 100, 150, and 200 °C and prepared. The sample preparation process and equipment configuration are shown in Fig. 1.

### SPS-CP

The pellet preparation process for SPS sintering was carried out in the same method as above. The sample was sintered at 1400 °C for 10 min under 40 MPa.

### GPS-CP

For GPS sintering that does not include a mold in the equipment, a specimen was first prepared through uniaxial press molding. YAG:Ce^3+^ powders were loaded into cylindrical mold, and compressed in a uniaxial press. The pellet molded into a cylinder shape was compressed under 300 MPa with a cold isostatic press, and then sintered at 1500 °C under 50 MPa for 6 h.

Samples prepared by each of the three methods were annealed at 1300 °C for 10 h in an air atmosphere for decoloring and carbon removal.

Luminous properties were measured by double integrating spheres (PSI Co., Ltd., Korea) under blue laser of excitation wavelength 445 nm. The internal porosity measurement of CPs was performed by AutoporeIV analyzer (Micromeritucs, USA). Surface morphology of samples was measured by field emission scanning electron microscopy (FE-SEM, JEOL, JSM-7600F). For cross-sectional SEM analysis, specimens were cut using a metal blade.

## Results and discussion

**Figure 2:**
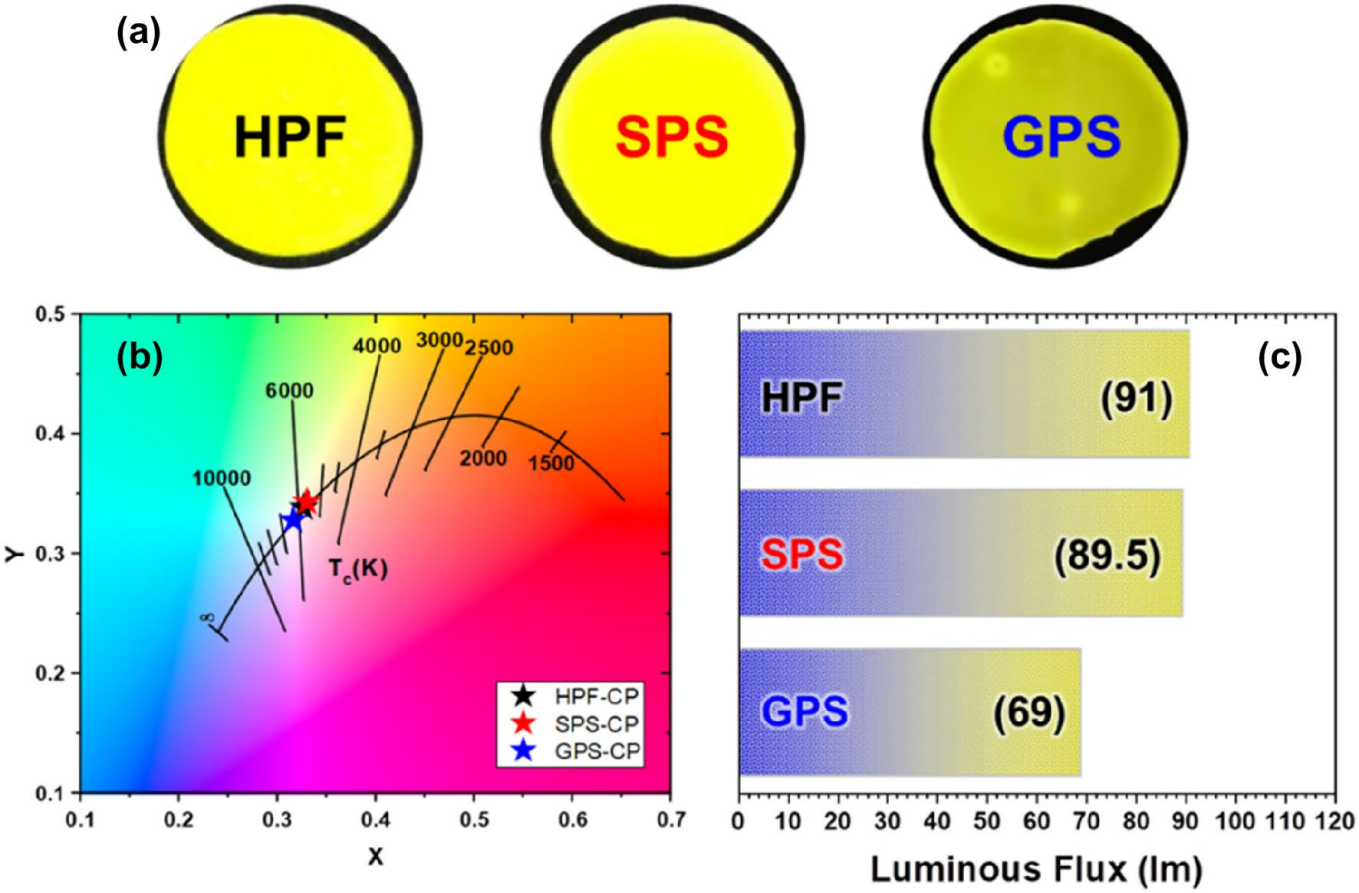
(**a**) Photographs of the CPs prepared by different method (from left to right: HPF, SPS, and GPS). (**b**,**c**) CIE color coordinates and luminous flux of CPs, respectively.

Figure 2 shows the optical properties for each HPF, SPS, and GPS that have different sintering methods. The fabricated samples show subtle differences, and it was confirmed that the samples generate white light that corresponds with the Planckian Locus. The Planckian Locus represents the Commission Internationale de l'Eclairage (CIE) color coordinates corresponding to the color temperature of 5500–6000 K of the automotive lighting regulations. However, Fig. 2a shows that despite the further polishing and post-annealing process, the CP fabricated by the GPS method shows a discolored surface. This result occurred because of the long sintering time compared with the other sintering methods. Due to the lower heating rate, the carbon from the GPS furnace that has carbon as a major component penetrated the deep region of the CP. As a result, Fig. 2b, shows that the GPS-sintered CP has a lower luminous flux of 69 lm, different from the other samples that have a luminous flux of 90 lm.

**Figure 3:**
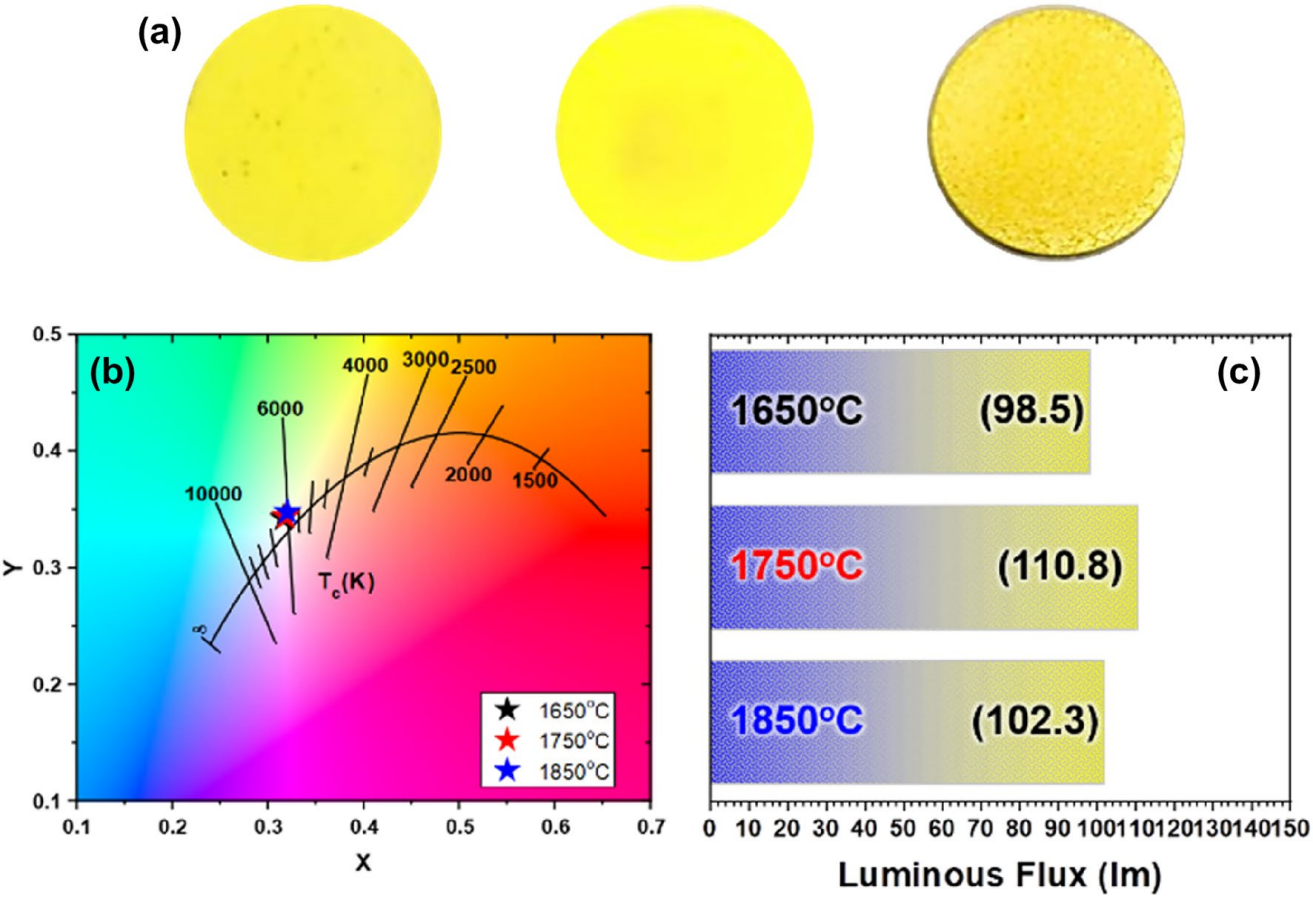
(**a**) Photographs of the CPs prepared by different sintering temperature based on HPF sintering (from left to right: 1650, 1750, and 1850 °C), SPS, and GPS). (**b**,**c**) CIE color coordinates and the luminous flux of CPs, respectively.

The potential of the HFP sintering method in the CP fabrication field has been partly proved by comparing the optical characteristics with conventional GPS and SPS methods. To optimize the factors that can affect performance, the CPs were fabricated by varying the sintering temperature, pressure, and heating rate. First, the sintering temperature was varied as 1650, 1750, and 1850 °C under the fixed condition with a heating rate of 150 °C/min and pressure of 50 MPa to investigate the performance trends. Figure 3a displays the photographs of fabricated CPs. Figure 3b shows that the CIE color coordinate of each sample shows similar trends in the white zone; however, different trend was clearly evident in the luminous flux. As confirmed in the graph of Fig. 3c, the result suggests that the CP that was sintered at 1750 °C exhibited the best luminous flux, and this result coincided with the color and surface condition of the CPs. The 1650-HFP sample has lower luminous flux due to the lower densification level comparing with the other temperature conditions, while the 1850-HFP exhibits the rough surface due to the coarsening of the particles as shown in Fig. 3a, inducing luminous flux degradation^28^.

**Figure 4:**
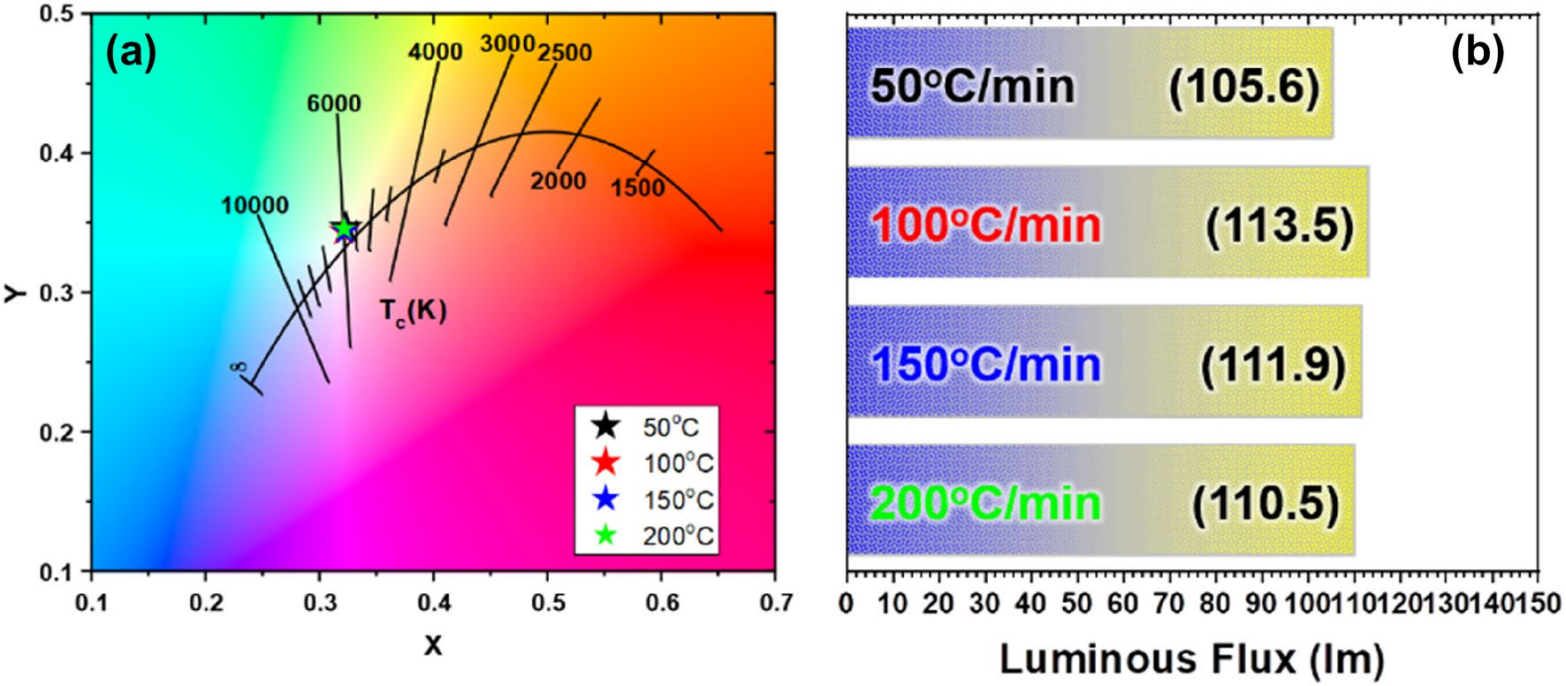
Optical properties of CPs prepared according to the heating rate in the range (50–200) °C.

**Figure 5:**
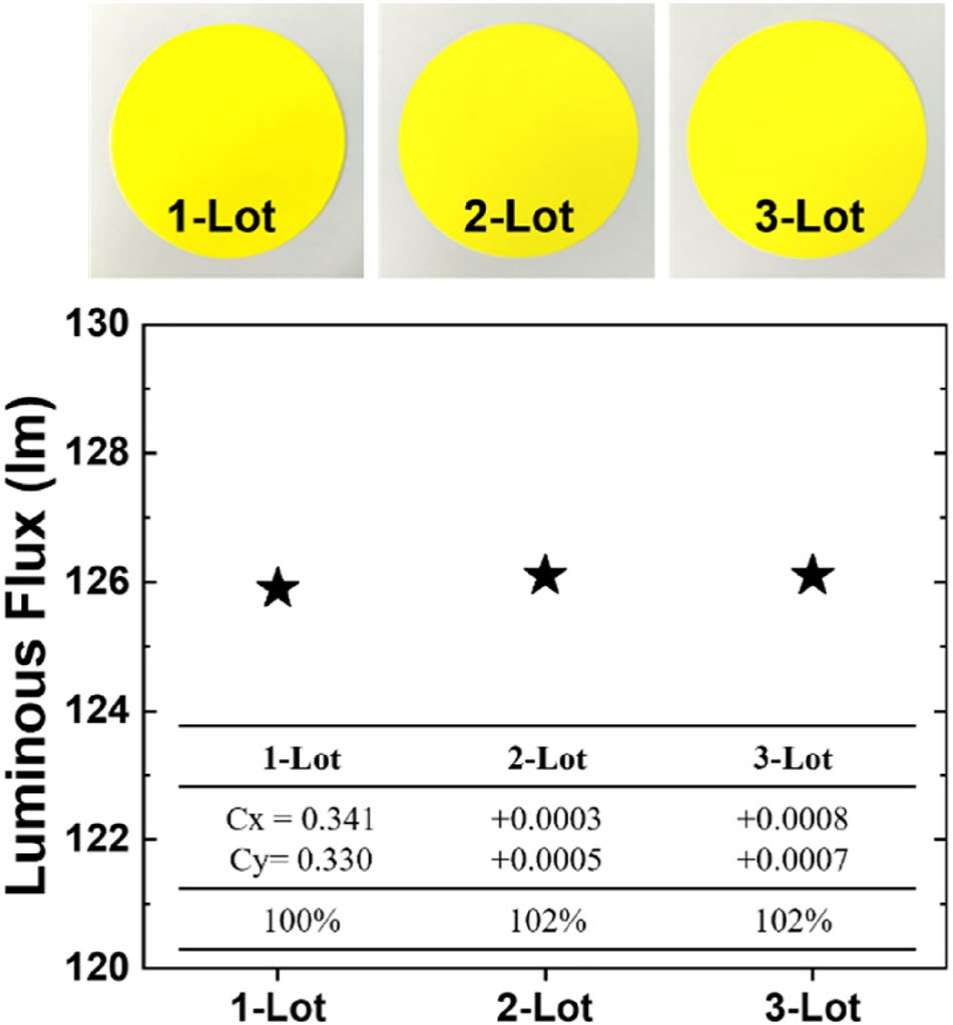
Reproducibility test of ceramic phosphor production using HFP sintering method.

To determine the effect of heating rate, the sintering process was varied as 50, 100, 150, and 200 °C/min at the optimized sintering temperature of 1750 °C. Figure 4a shows that the CIE color coordinates of the CPs that were fabricated under different heating rates did not exhibit obvious trends or differences between samples. In terms of luminous flux, the performance was improved on increasing the heating rate. However, Fig. 4b shows that the luminous flux improvement was saturated over 100 °C/min. When the CP was sintered at 50 °C/min heating rate, the coarsening was dominant over the grain boundary formation, and it led to less densification^27^. However, the coarsening rate and grain boundary formation rate were balanced over the 100 °C/min heating rate, and the optical performance was improved and saturated. The results imply that the superior condition is 100 °C/min, but the heating rates of 150 & 200 °C/min are also adaptable by considering producibility in the industry, because the performance difference is negligible. To confirm the reproducibility of the ceramic phosphor production, it was manufactured three times and the differences in the characteristics of the luminous flux and color coordinates were compared. As can be seen in Fig. 5, all three ceramic phosphors manufactured under the same conditions showed similar body color and optical properties. The derived results proved the performance of the HPF equipment not only in excellent productivity but also in reproducibility.

**Figure 6:**
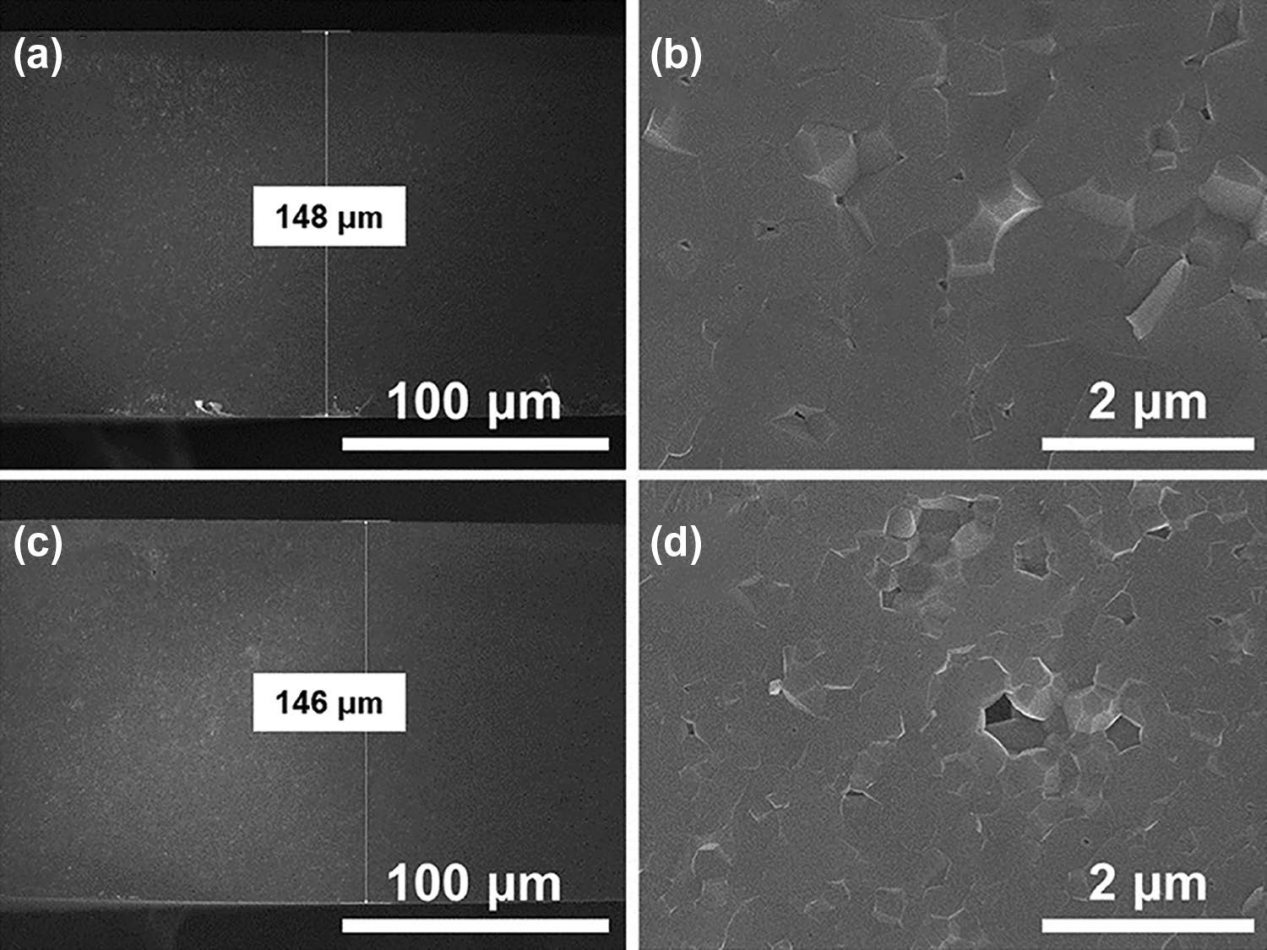
Cross-sectional SEM images of a CP fabricated under different pressure conditions. (**a**,**b**) zero MPa, (**c**,**d**) 50 MPa.

Figure 6 shows cross-sectional images of the fabricated CP to compare the densification depending on the presence of pressure during sintering. When the sample was sintered without pressure during sintering, only particle coarsening occurred. (Fig. 6a,b; 1750 °C, and 100 °C/min). Otherwise, it was confirmed that in the sample sintered under the same conditions except for periodically applying a pressure of 50 MPa (depicted in Fig. 6c,d, coarsening of the phosphor particles was suppressed, and more grain boundaries were formed. By optimizing the conditions, such as the sintering temperature, temperature increase rate, and pressurization of the HFP sintering method, it was possible to induce the formation of grain boundaries and improve the luminous flux. However, as shown in Fig. 6d, it was considered that the introduction of a post-treatment process was necessary because there were still residual large pores.

**Figure 7:**
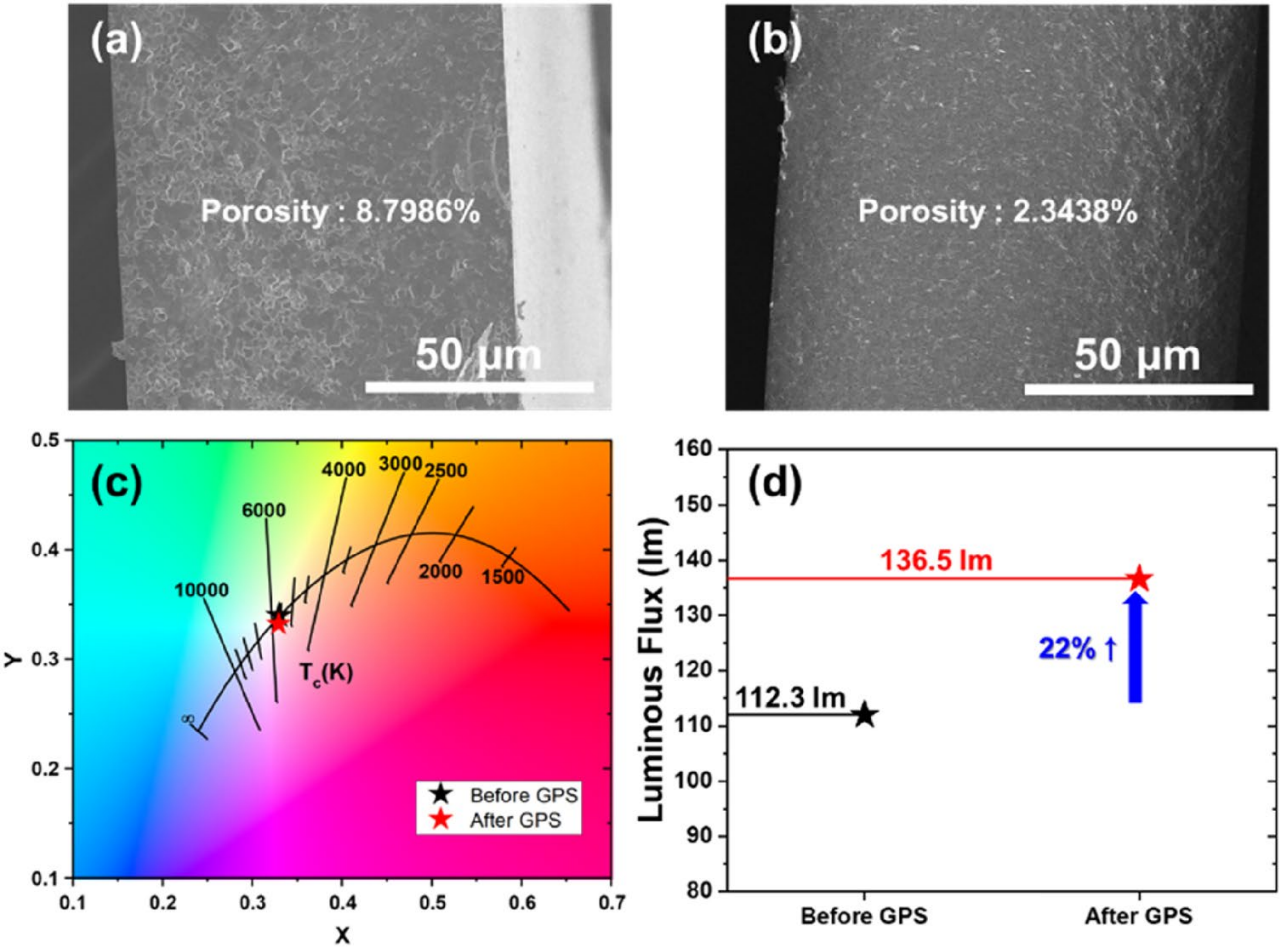
(**a**,**b**) Cross-sectional SEM images of the CPs with and without GPS post-processing, respectively, and (**c**,**d**) their optical properties before and after GPS.

To further improve the optical properties, additional GPS sintering was processed to remove residual pores in the CP. Figure 7a of the SEM image of the CP after HFP sintering shows a pore ratio of 8.7968% by showing pores between grains. Figure 7b shows that after additional GPS sintering, the pores were decreased, and the pore ratio was also reduced to 2.3438%. Figure 7c,d display the optical properties before and after GPS treatment. In Fig. 2a, it was mentioned that the GPS sintering method has the disadvantage of causing strong carburization inside the sintered body. However, since pore control of ceramics is a significant factor in laser applications, it is considered that when the GPS sintering method is adopted as a post-process, it can provide excellent performance in raising the sample quality. The CIE color coordinate was changed slightly, while the luminous flux exhibited a notable improvement of 22%. This result originated from the decrement of scattering in the plate through the reduced pore ratio^29^.

**Figure 8:**
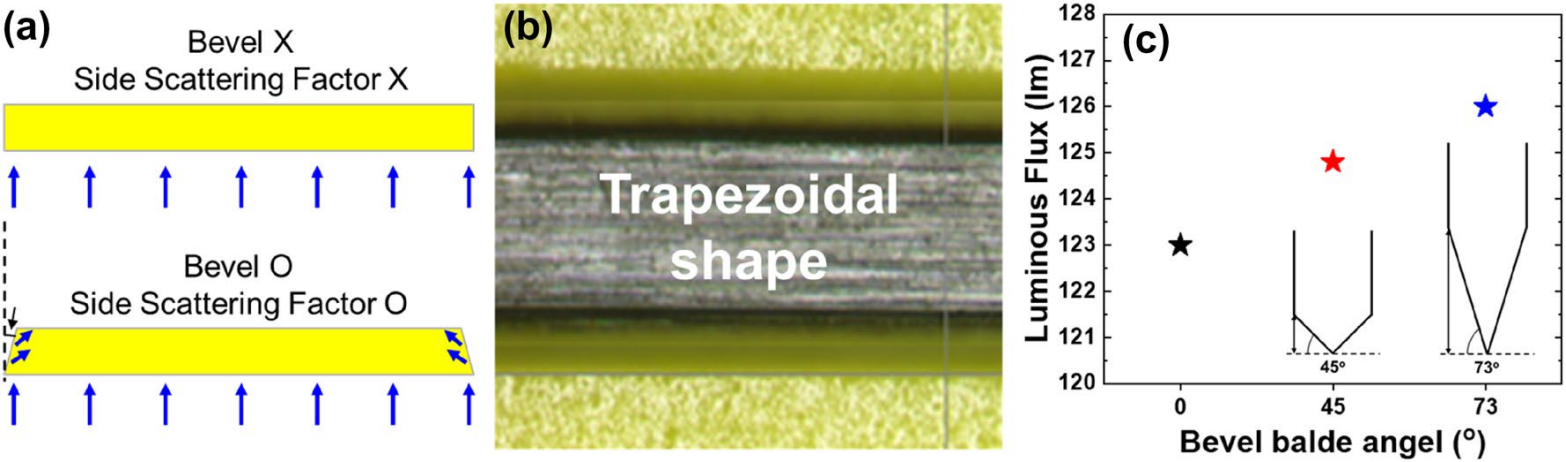
(**a**) schematic diagram of the effect of bevel cutting, (**b**) an optical microscope top view image of the bevel-cut phosphor ceramic, and (**c**) luminous flux with each bevel angle (0, 43, 73°).

Figure 8 shows the results of ceramic phosphors processed using beveled blades. The cutting process of the ceramic phosphor determines the shape of the square plate sidewalls. Depending on the shape of the blade used for cutting, a flat surface or an inclined surface can be obtained. The amount of cone-shaped light that is converted to the optical converter and emitted can be improved through random reflection by the trapezoidal inclined side (depicted in Fig. 8a)^30^. As shown in Fig. 8b, the processed ceramic phosphor shows inclined sidewalls. Figure 8c shows the measured luminous flux of the samples processed with a blade for each 0, 43, and 73° inclination angle, respectively. As a result, the luminous flux value of the ceramic phosphor cut with a 73° bevel blade was improved by about 2.4% compared to the ceramic phosphor cut flat.

## Summary

In summary, we investigated the optical properties of CPs fabricated by different sintering processes of GPS, SPS, and HPF. The HPF sintering conditions for high-quality YAG:Ce^3+^ CPs were controlled through three variables of sintering temperature, presence of pressure, and heating rate. As a result, the CP sintering condition was optimized to 1750 °C, 50 MPa, and 100 °C/min, and the CP that was fabricated by the optimized method shows a luminous flux of 114 lm and CIE color coordinate of (0.3216, 0.3439). Further post-processing of the GPS leads to an improvement of luminous flux of 22%. HPF-CP exhibits superior performances to GPS-CP, and comparable optical properties to the conventional SPS-CP. Even in terms of the heating rate, the HPF method has the advantage of improving the fabrication process. Considering the reliability, productivity, we suggest that the HFP sintering process could be a good candidate in the CP market.

## Data Availability

The data that support the findings of this study are available within the article.
